# A randomised controlled phase II trial of pre-operative celecoxib treatment reveals anti-tumour transcriptional response in primary breast cancer

**DOI:** 10.1186/bcr3409

**Published:** 2013-04-08

**Authors:** Rita D Brandão, Jürgen Veeck, Koen K Van de Vijver, Patrick Lindsey, Bart de Vries, Catharina HMJ van Elssen, Marinus J Blok, Kristien Keymeulen, Torik Ayoubi, Hubert JM Smeets, Vivianne C Tjan-Heijnen, Pierre S Hupperets

**Affiliations:** 1 Department of Clinical Genetics, Unit of Clinical Genomics, Maastricht University Medical Centre+, P. Debyelaan 25, Maastricht, 6229 HX, The Netherlands; 2 GROW - School for Oncology and Developmental Biology, Maastricht University Medical Centre+, P. Debyelaan 25, Maastricht, 6202 AZ, The Netherlands; 3 Department of Internal Medicine, Division of Medical Oncology, Maastricht University Medical Centre+, P. Debyelaan 25, Maastricht, 6229 HX, The Netherlands; 4 Department of Pathology, Maastricht University Medical Centre+, P. Debyelaan 25, Maastricht, 6229 HX, The Netherlands; 5 Institute of Pathology, RWTH Aachen University Hospital, Pauwelsstr. 30, Aachen, 52074, Germany; 6 Department of Internal Medicine, Division of Haematology, Maastricht University Medical Centre+, P. Debyelaan 25, Maastricht, 6229 HX, The Netherlands; 7 Pharmacell BV, Oxfordlaan 70, Maastricht, 6229 EV, The Netherlands; 8 Department of Surgery, Maastricht University Medical Centre+, P. Debyelaan 25, Maastricht, 6202 AZ, The Netherlands; 9 Saint James School of Medicine, Basic Science and Premedical Faculty, Kralendijk, Bonaire, Netherlands Antilles

## Abstract

**Introduction:**

Cyclooxygenase-2 (COX-2) is frequently over-expressed in primary breast cancer. In transgenic breast cancer models, over-expression of COX-2 leads to tumour formation while COX-2 inhibition exerts anti-tumour effects in breast cancer cell lines. To further determine the effect of COX-2 inhibition in primary breast cancer, we aimed to identify transcriptional changes in breast cancer tissues of patients treated with the selective COX-2 inhibitor celecoxib.

**Methods:**

In a single-centre double-blind phase II study, thirty-seven breast cancer patients were randomised to receive either pre-operative celecoxib (400 mg) twice daily for two to three weeks (*n *= 22) or a placebo according to the same schedule (*n *= 15). Gene expression in fresh-frozen pre-surgical biopsies (before treatment) and surgical excision specimens (after treatment) was profiled by using Affymetrix arrays. Differentially expressed genes and altered pathways were bioinformatically identified. Expression of selected genes was validated by quantitative PCR (qPCR). Immunohistochemical protein expression analyses of the proliferation marker Ki-67, the apoptosis marker cleaved caspase-3 and the neo-angiogenesis marker CD34 served to evaluate biological response.

**Results:**

We identified 972 and 586 significantly up- and down-regulated genes, respectively, in celecoxib-treated specimens. Significant expression changes in six out of eight genes could be validated by qPCR. Pathway analyses revealed over-representation of deregulated genes in the networks of proliferation, cell cycle, extracellular matrix biology, and inflammatory immune response. The Ki-67 mean change relative to baseline was -29.1% (*P *= 0.019) and -8.2% (*P *= 0.384) in the treatment and control arm, respectively. Between treatment groups, the change in Ki-67 was statistically significant (*P *= 0.029). Cleaved caspase-3 and CD34 expression were not significantly different between the celecoxib-treated and placebo-treated groups.

**Conclusions:**

Short-term COX-2 inhibition by celecoxib induces transcriptional programs supporting anti-tumour activity in primary breast cancer tissue. The impact on proliferation-associated genes is reflected by a reduction of Ki-67 positive cells. Therefore, COX-2 inhibition should be considered as a treatment strategy for further clinical testing in primary breast cancer.

**Trial registration:**

ClinicalTrials.gov NCT01695226.

## Introduction

Cancer development is associated with chronic immune activation, but the mechanisms behind this observation are not fully understood [1,2]. In addition, the inflammatory processes that follow tumour formation provide a microenvironment in which the development of malignant disease may be enhanced [3]. The involvement of chronic immune activation has been supported by several lines of evidence in which an association between non-steroidal anti-inflammatory drug (NSAID) consumption and decreased risk of cancer development, including breast cancer, has been demonstrated [4-11]. However, the exact mechanisms by which NSAIDs exert inhibiting effects on tumour development have not yet been completely elucidated.

Mediators of inflammatory responses, such as the cyclooxygenase (COX)-derived prostaglandins (PG), play an important role in tumour formation and provide a target for therapeutic intervention [3]. PGs have important functions in every organ system and regulate a variety of physiological functions, such as immunity, maintenance of vascular integrity and bone metabolism [12]. Elevated COX expression in breast cancer was first suggested by the finding of elevated PG production in breast cancer cells [13]. To date, two different COX genes have been characterised, *COX-1 *and *COX-2. COX-1 *is constitutively expressed by almost all human cells and, therefore, differs from *COX-2 *expression which is normally absent but is inducible by a wide spectrum of growth factors, pro-inflammatory cytokines [14,15] and tumour-promoting compounds [16,17]. Consistently, *COX-2 *is abundantly expressed in breast cancer tissue [18] and its enforced over-expression in mammary gland epithelia of transgenic mice results in breast tumour development [19], suggesting that COX-2 might be an interesting therapeutic target in breast cancer.

While several pre-clinical studies have indeed shown anti-tumour capacities of COX-2 inhibition, the treatment effects on primary breast cancer in the clinical setting remain elusive. Therefore, we aimed to determine for the first time transcriptional changes in primary breast cancer tissue of women with early breast cancer after treatment with the selective COX-2 inhibitor celecoxib. In our randomised controlled trial we found that upon celecoxib treatment numerous genes are differentially expressed in breast cancer tissues with an overall anti-tumour activity, suggesting that COX-2 inhibition should be further considered for clinical testing as a treatment option in breast cancer.

## Methods

### Patients and study design

**Figure 1:**
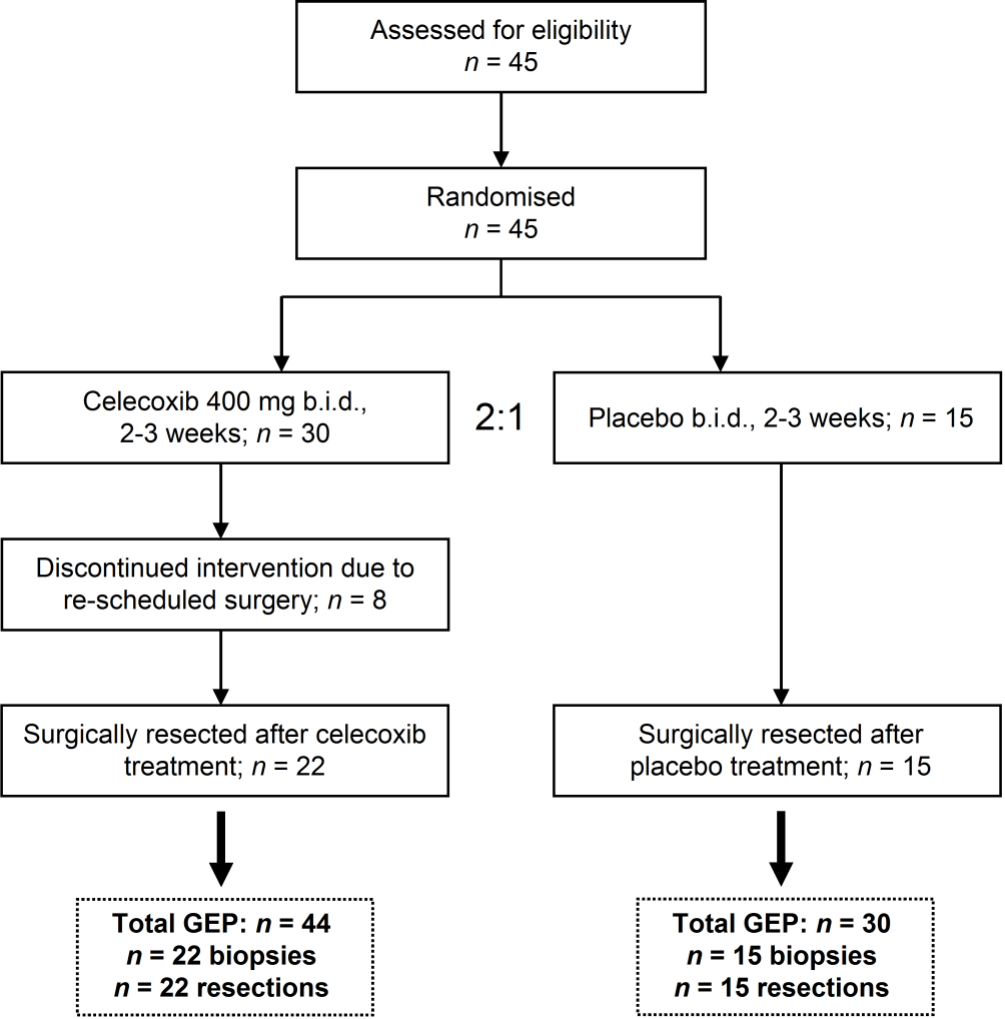
**Flow diagram of the presented study**. The design is a double-blind, randomised, controlled phase II trial of pre-operative celecoxib versus placebo in early breast cancer. Note that eight patients had discontinued intervention in the treatment arm. Gene expression profiling (GEP) has been performed from samples where indicated.

The study was a double-blind, randomised, placebo-controlled phase II pre-surgical trial of celecoxib in early breast cancer. Exclusion criteria were: HIV, hepatitis B virus (HBV) or hepatitis C virus (HCV) positivity, known hypersensitivity to NSAIDs, patients already using NSAIDs or systemic use of corticosteroids. Informed consent was obtained prior to entering the trial and the Medical Ethics Committee of the Maastricht University Medical Centre+ (MUMC+) approved the study. We estimated that to test 18,500 genes at the 5% significance level and ensure 80% power, 23 samples were needed to detect differentially expressed genes by t-tests with a fold change of at least 1.5 (http://bioinformatics.mdanderson.org/MicroarraySampleSize/). Initially, 45 patients were recruited between 2005 and 2007 and randomly allocated 2:1 to the treatment (*n *= 30) or placebo group (*n *= 15). Celecoxib was pre-surgically administered for two to three weeks at 400 mg twice daily, whereas patients in the control arm received a placebo on the same schedule. Eight patients allocated to the treatment arm dropped out because these patients were operated earlier, thus drug compliance was insufficient (Figure 1). Tumour histology was assessed according to criteria defined by the World Health Organization (WHO) [20], while staging was performed according to the Union for International Cancer Control (UICC) criteria [21]. Tumours were graded following the system of Bloom and Richardson, as modified by Elston and Ellis [22]. Patient characteristics are described in Table 1. Importantly, in our study design patients acted as their own control, with a direct comparison of the final surgical specimen with the initial diagnostic biopsy. The inclusion of a placebo group served to observe a possible confounding impact of the disease and the experimental procedure, thereby allowing determination of a differential impact of celecoxib only.

**Table 1:** Clinico-pathological characteristics of breast cancer patients.

Variable	Categorisation	Control	Celecoxib
		**Number = 15**	**Number = 22**

Mean age ± SD (years)^a^		57 ± 12	51 ± 12
Histological tumour type^b^			
	Invasive ductal carcinoma	13 (87%)	19 (86%)
	Invasive lobular carcinoma	2 (13%)	3 (14%)
Tumour size^c^			
	pT1	10 (67%)	8 (36%)
	pT2	5 (33%)	13 (59%)
	pT3	0 (0%)	1 (5%)
Lymph node status^c^			
	Negative (pN0)	11 (73%)	10 (45%)
	Positive (pN1-3)	4 (27%)	12 (55%)
Tumour grade^d^			
	G1	5 (33%)	1 (5%)
	G2	4 (27%)	7 (32%)
	G3	6 (40%)	14 (63%)
Oestrogen receptor status^e^			
	ER positive	11 (73%)	19 (86%)
	ER negative	4 (27%)	3 (14%)
Progesterone receptor status^e^			
	PgR positive	10 (67%)	17 (77%)
	PgR negative	5 (33%)	5 (23%)
HER2 status^f^			
	HER2 positive	3 (20%)	3 (14%)
	HER2 negative	12 (80%)	18 (82%)
	Unknown	0 (0%)	1 (5%)

^a^At time of diagnosis; ^b^according to Tavassoli and Devilee [20]; ^c^according to Sobin and Wittekind [21]; ^d^according to Elston and Ellis [22]; ^e^positivity defined as > 10% positive cells as analysed by immunohistochemistry [60]; ^f^positivity defined by immunohistochemistry (score 3+) or by dual-color FISH. Percentages may not sum-up to 100 due to rounding. FISH, fluorescence *in situ *hybridisation.

### Biopsy method

Before patient allocation to the groups, two to three core needle biopsies from the centre of the primary tumour were obtained using a 14-gauge needle under ultrasound guidance. At surgery, a central sample of the excised tumour was obtained. One part of the biopsies and surgical excision specimens was snap-frozen in liquid nitrogen within 30 minutes after removal and stored at -80°C until use, while the remaining part was formalin-fixed and paraffin-embedded (FFPE). All specimens subjected to gene expression analyses had a tumour cellularity of at least 80%, as determined by haematoxylin and eosin stained sections.

### RNA isolation, cRNA production and fragmentation, array hybridisation and scanning

After homogenisation of fresh-frozen tissue specimens, total RNA was isolated using TRIzol reagent (Invitrogen, Carlsbad, CA, USA) according to the manufacturer's protocol. DNase treatment was performed with the RNase-Free DNase Set (Qiagen, Hilden, Germany) followed by purification of the RNA samples using the RNeasy Minikit (Qiagen). RNA quantity and purity were determined spectrophotometrically using the Nanodrop ND-1000 (Nanodrop Technologies, Wilmington, DE, USA) and RNA integrity was assessed by determining the RNA 28S/18S ratio using the Bioanalyzer 2100 (Agilent Technologies, Santa Clara, CA, USA). Biotinylated aRNA was synthesised and fragmented using the GeneChip IVT Express Kit from Affymetrix (Santa Clara, CA, USA). Hybridisation to Affymetrix Human Genome U133 Plus 2.0 arrays and subsequent scanning was performed following the manufacturer's guidelines using the GeneChip scanner 3000 (Affymetrix). Microarray datasets are publicly available at ArrayExpress database [23] under accession number E-MTAB-566.

### Microarray data analysis

Images of the arrays were quantified with GCOS software (Affymetrix). The chip description file (CDF) used for the analysis was an update created and freely distributed by the microarray lab of the University of Michigan [24] based on Ensembl (version 10). The treatment effects on global gene expression were assessed by microarray analyses of subjects after celecoxib treatment versus subjects before treatment and controls (before and after treatment). Thus, expression changes have been adjusted for differences in the pre- and post-treatment specimens of the control group. The genes were analysed using a Gaussian linear regression including the hybridisation and labelling spikes and *COX-2*. The inference criterion used for comparing the models is their ability to predict the observed data, that is, models are compared directly through their minimised minus log-likelihood. When the numbers of parameters in models differ, they are penalised by adding the number of estimated parameters, a form of the Akaike information criterion (AIC) [25]. For each gene, the group effect only after intervention was then added to the model. The gene under consideration was found to be differentially expressed if the AIC decreased compared to the model not containing this effect. All statistical analyses presented were performed using the freely available program R [26] and the publicly available library 'growth' [27].

### Validation of microarrays by quantitative RT-PCR

Quantitative RT-PCR (qPCR) was performed for selected genes on all 74 samples in order to validate the results obtained in the microarray study. As a reference, TATA box binding protein (*TBP*) and RNA, 18S ribosomal 1 (*RN18S1*) were included as housekeeping genes. The list of genes and the primers that were used are described in Additional file 1, Table S1. Excess biotinylated aRNA (before the fragmentation step) was used for validation, after cDNA synthesis with M-MuLV Reverse Transcriptase (Finnzymes, Espoo, Finland) and oligo(dT), (Invitrogen) using the SensiMix SYBR Kit (Quantace, London, UK) following the manufacturer's protocol. qPCRs were run on the 7900HT system (Applied Biosystems, Singapore). Results were analysed using a Gaussian linear regression similar to microarray data. Expression of housekeeping genes (*TBP *and *GAPDH*) and *COX-2 *were included during the analysis. The AIC was used to assess whether there was a difference between the controls and patients (group effect).

### Analysis of functional categories

Significantly altered genes that were found to have a fold-change difference of at least 10% were classified into categories of biological processes and molecular functions using DAVID (Database for Annotation, Visualisation and Integrated Discovery) [28] and PathVisio [29]. DAVID and PathVisio analyses for pathway enrichment were performed first for all significantly altered genes, then for up- and down-regulated genes separately. DAVID results are listed together with *P*-values corrected with the Bonferroni method, as this was the most conservative method used by the software. A *P*-value of 0.05 was used as a cut-off value. In both analyses we filtered the pathways or gene ontology (GO) terms for which less than five and more than 150 genes were found, as those were either too specific or too general for our analysis. In PathVisio, the gene database Hs_Derby_20110601.bridge and the pathway collection from WikiPathways [30] were used to obtain a ranked list of pathways with differentially expressed genes. PathVisio results were sorted by *Z*-score, which is the standard statistical test under the hypergeometric distribution. Only pathways with a *Z*-score above 3, which corresponds to *P*-values of 0.0013 or lower, were selected.

### Tissue marker expression

Biomarkers of response were assessed by immunohistochemistry on FFPE tissues. Cell proliferation was assessed using the MIB1 mouse monoclonal antibody to Ki-67 [31]. Measurement of apoptosis was performed by cleaved caspase-3 (Asp175) staining [32]. Tumour cells/section were counted for Ki-67 (1,000 cells) and apoptotic index (3,000 cells). The apoptotic index was expressed as a percentage of the number of cells displaying apoptotic bodies, scoring 0 if < 0.5%, +1 if between 0.5% and 2%, and +3 if > 2%. Samples were also assessed for expression of CD34 [33] using the Chalkley method [34].

### Statistical analyses

Ki-67 values at baseline and time of surgery were expressed as geometric mean proportions of the baseline and transformed into percentage changes. Ki-67 changes within groups were compared using a paired t-test, differences in Ki-67 and CD34 between groups by using an unpaired t-test, and the Mann-Whitney test was used for group comparisons. Differences in caspase-3 expression were assessed by the Freeman-Halton extension of the Fisher's exact probability test. All tests were performed two-sided at the 5% significance level.

## Results

### Celecoxib treatment changes gene expression in breast carcinoma tissue

**Figure 2:**
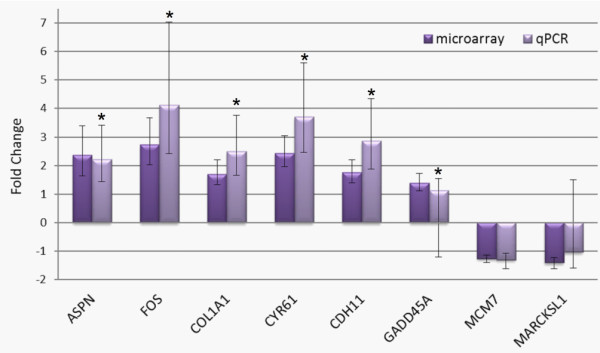
**qPCR validation of selected genes differentially expressed in celecoxib-treated samples as determined by microarray analysis**. Fold-change and the 95% CI (error bars) are shown. Expression of six out of eight genes analysed (indicated by asterisks) was significantly changed in agreement with the microarray analysis.

Two to three weeks of celecoxib treatment significantly altered the expression of 1,558 genes in breast cancer tissues, of which 972 genes were up- and 586 genes were down-regulated after treatment and adjustment to control tissue gene expression. The 50 most strongly up-regulated and down-regulated genes are presented in Additional file 2, Table S2 and Additional file 3, Table S3, respectively. For all of the selected genes, except two, significant expression changes were confirmed by qPCR (Figure 2).

The list of the biological pathways, in which significantly changed genes were over-represented, identified by DAVID analysis, is shown in Table 2. Biological processes identified by PathVisio are shown in Table 3. In both approaches down-regulated genes were consistently over-represented in cell cycle-related processes and proliferation. Up-regulated genes were over-represented in extracellular matrix (ECM) organisation, cell adhesion, and blood vessel development in DAVID analyses, whereas PathVisio results suggested an implication of further tumour suppressive pathways, for example, complement activation, senescence and autophagy, and transforming growth factor-β (TGF-β) signaling.

**Table 2:** Over-represented functional categories within genes up- and/or down-regulated after celecoxib treatment using DAVID.

GO-ID	Term	Count	Fold enrichment	***P-*value**^a^
**Up-regulated**				

0030198	Extracellular matrix organisation	28	4.657	6.35E-08
0009611	Response to wounding	70	2.337	1.32E-07
0007155	Cell adhesion	83	2.108	3.24E-07
0022610	Biological adhesion	83	2.105	3.46E-07
0001944	Vasculature development	43	2.951	1.20E-06
0001568	Blood vessel development	42	2.953	1.96E-06
0043062	Extracellular structure organisation	33	3.502	2.35E-06
0051270	Regulation of cell motion	34	3.047	4.89E-05
0001501	Skeletal system development	46	2.510	5.23E-05
0030334	Regulation of cell migration	31	3.173	9.15E-05
0040012	Regulation of locomotion	33	2.973	1.51E-04
0042127	Regulation of cell proliferation	83	1.834	2.27E-04
0042060	Wound healing	32	2.944	3.14E-04
0007160	Cell-matrix adhesion	20	3.931	1.42E-03
0048514	Blood vessel morphogenesis	33	2.692	1.56E-03
0031589	Cell-substrate adhesion	21	3.745	1.57E-03
0006928	Cell motion	53	1.984	7.97E-03
0001525	Angiogenesis	25	2.922	1.15E-02
0009612	Response to mechanical stimulus	14	4.324	3.91E-02

**Down-regulated**				

0006259	DNA metabolic process	52	3.018	5.10E-09
0007049	Cell cycle	64	2.411	1.85E-07
0006260	DNA replication	28	4.387	3.98E-07
0022402	Cell cycle process	50	2.569	4.12E-06
0000278	Mitotic cell cycle	37	2.898	3.70E-05
0000279	M phase	32	2.832	6.92E-04
0051276	Chromosome organisation	40	2.461	7.18E-04
0006974	Response to DNA damage stimulus	34	2.656	1.20E-03
0000087	M phase of mitotic cell cycle	25	3.229	1.76E-03
0022613	Ribonucleoprotein complex biogenesis	22	3.544	2.07E-03
0000280	Nuclear division	24	3.156	4.53E-03
0007067	Mitosis	24	3.156	4.53E-03
0022403	Cell cycle phase	35	2.454	4.81E-03
0042254	Ribosome biogenesis	17	4.062	8.27E-03
0048285	Organelle fission	24	3.031	9.01E-03
0006281	DNA repair	27	2.785	9.53E-03
0006396	RNA processing	41	2.185	1.01E-02
0031396	Regulation of protein ubiquitination	15	4.434	1.30E-02
0031397	Negative regulation of protein ubiquitination	13	5.177	1.31E-02
0031145	Anaphase-promoting complex-dependent proteasomal ubiquitin-dependent protein catabolic process	12	5.461	2.01E-02
0016071	mRNA metabolic process	31	2.428	2.52E-02
0034470	ncRNA processing	20	3.133	4.28E-02
0051439	Regulation of ubiquitin-protein ligase activity during mitotic cell cycle	12	4.986	4.85E-02

^a^*P-*values shown were corrected for multiple testing using the Bonferroni method; categories ranked by *P-*value; functional categories with *P-*values < 0.05 and at least five, but less than 150 genes meeting the criterion are shown.

**Table 3:** List of local pathways in PathVisio significantly affected by the celecoxib treatment.

MAPP Name	number changed	number measured	% changed	***Z*-score**^a^
**Up-regulated**				

Complement and coagulation cascades	15	50	30.00%	5.65
Senescence and autophagy	18	100	18.00%	3.61
RANKL/RANK signalling pathway	11	50	22.00%	3.58
Complement activation, classical pathway	5	15	33.33%	3.55
Matrix metalloproteinases	7	29	24.14%	3.14
TGF beta signalling pathway	18	113	15.93%	3.03
Inflammatory response pathway	7	30	23.33%	3.03
Oxidative stress	6	24	25.00%	3.01
Adipogenesis	19	125	15.20%	2.89
Endochondral ossification	11	61	18.03%	2.82
TWEAK signalling pathway	8	40	20.00%	2.73
SREBP signalling	6	28	21.43%	2.56

**Down-regulated**				

Cell cycle	17	95	17.89%	6.62
DNA replication	9	41	21.95%	5.61
Nucleotide metabolism	5	17	29.41%	5.12
Proteasome degradation	10	57	17.54%	4.97
Electron transport chain	10	81	12.35%	3.61
G1 to S cell cycle control	7	67	10.45%	2.51

^a^*Z*-score is the standard statistical test under the hypergeometric distribution; pathways ranked by *Z*-score. *Z*-score > 1.96 (corresponding to *P*-value = 0.05) and at least five, but less than 150 genes meeting the criterion are shown. TGF, tumour growth factor.

### Effects of celecoxib on cell cycle gene expression

**Figure 3:**
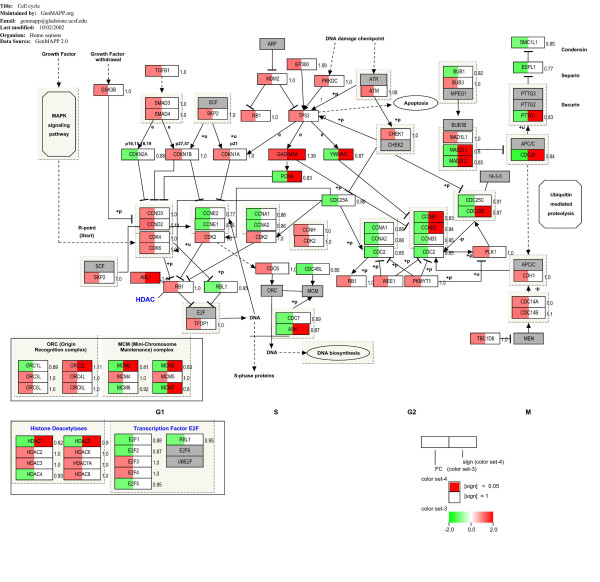
**Effects of celecoxib treatment on cell cycle and proliferation**. Contributed map from GenMAPP software with an overview of the pathways and genes involved in cell cycle regulation. The expression fold-changes of each gene are indicated next to the gene box. Genes highlighted in red and green in the left box half represent genes with fold-changes increased and decreased, respectively. Red colour in the right box half indicates a significant change. Grey boxes correspond to genes that were not analysed in the arrays.

DAVID and PathVisio consistently identified biological processes referring to regulation of cell cycle and proliferation, in particular among those genes down-regulated after treatment. An important downstream DNA damage response gene, *GADD45A *(Growth arrest and DNA-damage-inducible, alpha), was significantly up-regulated (Figure 3). Consistent with a putative activation of the G2/M checkpoint and cell cycle arrest due to DNA damage response, target genes *CCNB1 *(G2/mitotic-specific cyclin-B1) and *CCNB2 *(G2/mitotic-specific cyclin-B2) were significantly down-regulated after treatment.

### Effects of celecoxib on ECM degradation gene expression

**Figure 4:**
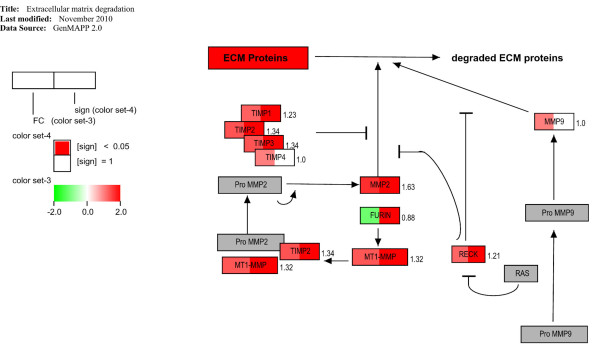
**Effects of celecoxib treatment on extracellular matrix protein degradation**. Map designed on GenMAPP software with an overview of the genes involved in the extracellular matrix protein degradation process. The expression fold-changes of each gene are indicated next to the gene box. Genes highlighted in red and green in the left box half represent genes with fold-changes increased and decreased, respectively. Red colour in the right box half indicates a significant change. Grey boxes correspond to genes that were not analysed in the arrays.

The majority of the matrix metalloproteinase (MMP) family members have been associated with tumour progression. The conversion of pro-MMP to active MMP-2 requires membrane type MT1-MMP (MMP-14), a trans-membrane protein that is activated intracellularly by the convertase FURIN [35]. The down-regulation of the protein convertase *FURIN *in the celecoxib-treated group potentially leads to less activation of MT1-MMP. Additionally, the effect of MMP-2 on proteolysis was inhibited either by up-regulation of *TIMP1, TIMP2, TIMP3*, or by *RECK *(Figure 4). The up-regulated *RECK *exerts inhibitory effects on the conversion of pro-MMP-2 to MMP-2 and on the activation of pro-MMP-9 to MMP-9. In summary, our data suggest that degradation of ECM proteins was significantly inhibited in the celecoxib-treated group.

### Recruitment of tumour-infiltrating leukocytes to breast carcinoma tissue after celecoxib treatment

Breast cancer tissue of celecoxib-treated patients showed a significantly increased expression of MHC class II genes, including *HLA-DRα *and *HLA-DRβ2, CD74 *(MHC class II invariant chain) and *HLA-DM*, but not *HLA-DQ *and *HLA-DOA *[see Additional file 4, Table S4]. MHC class I gene expression was not significantly changed. Subsequent to the increased expression of HLA-class II genes, co-stimulatory markers of antigen-presenting cells (*CD83*) and the monocyte differentiation antigen *CD14 *were up-regulated after celecoxib treatment. Infiltration of antigen-presenting cells was supported by increased expression of the Pattern Recognition Receptors (PRR), Toll-like receptor-2 (*TLR2*) and MD-1 (*LY86*). Gene expression of the classical B cell markers, *CD20 *and *CD19*, was not altered, although there was increased expression of immunoglobulin J chain. As myeloid-derived suppressor cells (MDSC) immune suppressive cells also express MHC class II molecules, and their presence correlates with COX-2 over-expression [36], induction of MDSC signalling was investigated. However, MDSC induction seems unlikely, since the expression of MDSC-signalling genes *ARG1 *and *NOS2 *is not altered. Moreover, expression of important effector molecules, such as granzymes and perforin, was not affected. The increased infiltration of leukocytes observed in the breast tumour seems restricted to macrophages and dendritic cells.

### Change of tissue biomarker expression

**Figure 5:**
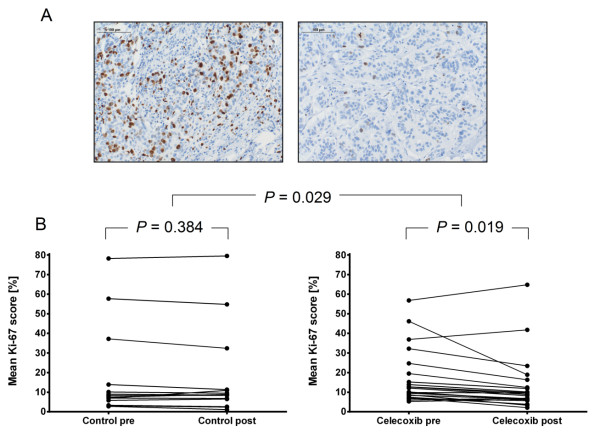
**Effects of celecoxib treatment on Ki-67 protein expression**. **(A) **Examples of immunohistochemical staining of nuclear Ki-67 protein on breast cancer tissues yielding a high score (left) and low score (right). Scale bar = 100 μm. **(B) **Shown are the Ki-67 scores from individual patients in the control arm (plot on left-hand side) and treatment arm (plot on right-hand side). Geometric means in the control group were statistically not different (*P *= 0.384), while the geometric mean after celecoxib treatment was significantly reduced (*P *= 0.019). Also, the change of the means between both groups was significantly greater in the celecoxib-treated group (*P *= 0.029).

To confirm the transcriptional changes, we determined expression of protein markers for proliferation, apoptosis, and neo-angiogenesis. The proliferation marker Ki-67 was assessed on paired pre- and post-treatment tissues. Due to a lack of further tissue, apoptotic marker cleaved caspase-3 and neo-angiogenesis marker CD34 were assessed only on post-treatment tissues. Baseline Ki-67 positivity in the control group (geometric mean: 10.0%; 95% CI: 5.5 to 18.3) was not significantly different from baseline Ki-67 positivity in the treatment group (geometric mean: 13.4%; 95% CI: 9.8 to 18.3) (*P *= 0.915). The change in Ki-67 is shown for individual patients according to treatment or control arm in Figure 5. The geometric mean change in Ki-67 relative to baseline in the treatment arm was -29.1% (95% CI: -40.1% to -16.2%, *P *= 0.019), whereas in the control arm it was -8.2% (95% CI: -23.1 to 9.5%, *P *= 0.384). There was a significant change difference between the two groups (*P *= 0.029). In contrast, the apoptotic index was not significantly different in post-treatment tissues (*P *= 0.231). The number of CD34 positive cells was slightly higher in celecoxib-treated tissues, but this was statistically insignificant. The geometric means (95% CI) of the 'Chalkley mean' value were 6.8 (5.4 to 8.5) in the control group and 7.7 (6.8 to 8.8) in the treatment group (*P *= 0.376).

## Discussion

In this study, we analysed the transcriptional changes seen in primary breast cancer tissue following short-term celecoxib treatment. To accomplish this, we used global gene expression profiles from paired pre- and post-treatment specimens. After adjustment to the control group, we identified a large number of differentially expressed genes after treatment that are involved in the regulation of cancer-associated pathways, such as cell cycle and proliferation, ECM biology, and inflammatory response, amongst others. Most convincingly, COX-2 inhibition induced gene expression patterns indicative of a decelerated cell cycle and reduced proliferation. Celecoxib may induce G2/M arrest by p53 activation, leading to *GADD45A *up-regulation, which in turn inhibits cyclin-B1 and cyclin-B2 expression and promotes G2/M arrest (Figure 3). A G2/M arrest is mainly forced after DNA damage to enable the initiation of DNA repair mechanisms [37]. Our finding is in line with previous studies investigating the effects of celecoxib on cancerous cells *in vitro*. Dvory-Sobol and colleagues demonstrated that celecoxib induces G2/M arrest associated with cyclin-B1 down-regulation in *K-RAS*-transformed enterocytes [38], and in the COX-2 expressing murine breast cancer cell line MCa-35, celecoxib induced a G2/M arrest followed by apoptosis [39]. Interestingly, equally treated lung cancer A549 cells lacking COX-2 expression showed increased DNA damage, but low levels of apoptosis in these cells suggested a selective effect of celecoxib on COX-2 expressing cells [39]. Celecoxib seems to increase DNA damage in irradiated cells, enhancing their radiosensitivity [40]. However, the mechanisms behind increased DNA damage in celecoxib-treated tumour cells remains poorly understood. Our results are further consistent with a study of colorectal cancer (CRC) cell lines in which more than 1,000 genes were identified as differentially expressed after celecoxib treatment, clustering of which revealed significant changes of cell cycle control, apoptosis, and lymphocyte infiltration [41]. Also in a study on primary CRC, celecoxib-induced gene expression changes significantly interfered with proliferation pathways [42]. In summary, we have confirmed a positive treatment effect of COX-2 inhibition on cell proliferation-related transcriptional programs in primary breast carcinomas, as has been previously demonstrated by several *in vitro *and *in vivo *studies.

Disruption of the basement membrane is a hallmark of malignancy. Degradative enzymes, such as MMPs, are produced by tumour cells and by resident and infiltrating cells as a response to the tumour, and contribute to matrix degradation and facilitate tumour invasion. MMP-2, MMP-9, and other members of the MMP family have been associated with tumour progression [43]. In particular, MMP-2 and MMP-9 activity appears to be inhibited by celecoxib in our study, the first by up-regulation of MMP antagonists (*TIMP1, TIMP2*, and *TIMP3*), the latter by up-regulation of the MMP-9 inhibitor *RECK*. An involvement of selective COX-2 inhibition in matrix stability by decreasing MMP activity and tumour invasiveness has been previously demonstrated in breast and CRC cancer models [44,45], thus being in good agreement with our data.

Several lines of evidence demonstrated that immune cell infiltration in tumours is enhanced by celecoxib treatment, which is associated with a better prognosis [46,47]. In our study, increased infiltration of antigen-presenting cells is supported by gene expression data whereas other immune cells of both the innate and adaptive immune system do not seem to be affected by celecoxib treatment. Most up-regulated genes within this category belong to MHC class II. Comparable data on MHC class I and II induction have been reported by Lönnroth *et al*. in CRC patients using a NSAID [47].

In order to investigate whether observed gene expression changes after COX-2 inhibition have translated to a biologically relevant effect, we analysed protein markers for proliferation, apoptosis, and neo-angiogenesis in primary tissues. Suppression of the proliferation marker Ki-67 has been previously reported as a surrogate marker for decreased aromatase activity in oestrogen receptor (ER) positive breast cancer treated with aromatase inhibitors (AI) for two weeks [48,49]. Since COX-2 expression is positively correlated with tumour aromatase content [50], we were interested whether COX-2 inhibition would also lead to a reduction in Ki-67 positivity. As expected from previous studies [51,52], Ki-67 was not significantly reduced in the control arm. In contrast, the celecoxib arm showed a significant suppression of Ki-67, confirming the reduced proliferation observed in our gene expression data and suggesting an indirect treatment effect on aromatase activity. Although the Ki-67 suppressive effect was only modest (-29%) as compared to the AI anastrozole (-75%) [49], it was similar to the Ki-67 suppression achieved with another AI, that is, raloxifene (-24%) [52]. Other than Ki-67, caspase-3 and CD34 were not significantly changed after celecoxib treatment, although this has to be interpreted cautiously due to the lack of baseline data. However, in a previous neo-adjuvant study in breast cancer, two weeks of celecoxib did not result in a biological response of proliferation and apoptosis, as determined by Ki-67 staining and TUNEL assays, respectively [53]. Notably, the referenced study analysed fewer patients and used half the drug dose that we applied. Taken together, we hypothesise that two weeks of COX-2 inhibition may not be sufficient to translate all transcriptional activation to a measurable biological phenotype. In particular, suppression of blood vessel development may take effect only after longer drug exposure, which should be taken into consideration when designing future clinical trials of COX-2 inhibition in cancer.

In breast cancer, COX-2 over-expression is positively associated with HER2 over-expression [54] and with tumour aromatase content [50]. Therefore, COX-2 inhibition might prove beneficial, especially in combination with trastuzumab in HER2 positive breast cancer or with AI in hormone receptor (HR) positive disease. In trastuzumab-refractory metastatic breast cancer COX-2 inhibition was previously shown to be inactive [55]. However, improved efficacy and endpoint benefits of celecoxib in combination with AI were reported in post-menopausal metastatic breast cancer, although these were pronounced only in tamoxifen resistant patients [56,57]. Although promising, further studies are needed in order to elucidate a clinical benefit of COX-2 inhibition in combination with other drugs in breast cancer treatment.

The strength of our study is the trial design, which allows patients to act as their own control, and the inclusion of a placebo group, which served to exclude potentially confounding effects by the disease and the experimental procedures. It would be interesting to sub-analyse the gene expression and Ki-67 in stratified breast cancer subtypes, as for example, HR positive *versus *negative disease, but this is hindered by the low number of HR negative patients in the control (*n *= 5) and the treatment arm (*n *= 1). Of note, however, there is a trend of bias towards poorer prognostic factors in the celecoxib arm, which could not have been predicted or changed before the end of the treatment procedure. Although this bias must be considered a limitation of the study, we speculate that the observed anti-tumour transcriptional response in this arm may have been under-estimated, and thus could become even more evident by comparing clinically highly similar arms.

Unfortunately, eight patients dropped out of the study due to early surgery, all of them from the treatment arm, resulting in a reduced statistical power to detect differentially expressed genes. However, in our microarray analysis we found more differentially expressed genes than expected by chance alone (1,558 observed versus 920 expected). After identification of the differentially expressed genes, pathway analyses were performed in order to identify genes clustering within the same biological pathway, as those genes are very likely to be true positives.

Large meta-analyses have recently shown that the non-selective COX inhibitor aspirin is protective against cancer development. Regular intake of aspirin significantly reduced the risk of several cancers, including breast cancer (OR = 0.88) [58], and three years onwards of daily aspirin intake reduced cancer incidence in both women and men (OR = 0.75) [59]. These studies suggest that selective COX-2 inhibitors may have similar effects on cancer incidence albeit with the advantage of causing less adverse side effects associated with aspirin use, such as gastrointestinal bleeding. COX-1 is expressed constitutively in many different tissues, whereas COX-2 is conditionally induced, such as in inflammation, suggesting that selective COX-2 inhibition might prove more successful in cancer prevention than non-selective COX inhibitors.

## Conclusions

Our clinical trial provides substantial evidence for an anti-tumour activity of celecoxib based on global transcriptional changes and suppression of Ki-67 protein in primary breast cancer tissues, encouraging further clinical trials of celecoxib or its derivative molecules in breast cancer. Our study confirms results from previous *in vitro *and *in vivo *studies as we found a large number of cell-cycle and proliferation-associated genes to be differentially expressed in celecoxib-treated primary breast cancer tissues. Our aim was to determine the global transcriptional response to short-term COX-2 inhibition in primary tumours, and our results indicate that proliferation and ECM biology are significantly influenced, possibly underlying the proposed anti-tumour activity. Future trials of COX-2 inhibition considering other primary endpoints, such as pathological or clinical response, should take into account that effects of a transcriptional response may need a longer time to translate into a measurable clinical benefit.

## Abbreviations

AI: aromatase inhibitor; AIC: Akaike information criterion; COX-2: cyclooxygenase-2; CRC: colorectal cancer; DAVID: Database for Annotation, Visualisation and Integrated Discovery; ECM: extracellular matrix; ER: oestrogen receptor; FFPE: formalin-fixed and paraffin-embedded; GEP: gene expression profiling; GO: gene ontology; HR: hormone receptor; MDSC: myeloid-derived suppressor cells; MMP: matrix metalloproteinase; NSAID: non-steroidal anti-inflammatory drug; PG: prostaglandin; qPCR: quantitative polymerase chain reaction; RT-PCR: reverse transcriptase polymerase chain reaction.

## Competing interests

The authors declare that they have no competing interests.

## Authors' contributions

RDB performed microarray data analyses, participated in qPCR validation, data interpretation and co-drafted the manuscript. JV participated in qPCR validation, data interpretation, statistical analyses of immunohistochemical results and co-drafted the manuscript. KKVdV performed pathological review and immunohistochemical scoring, participated in data interpretation and critically revised the manuscript. PL provided statistical analyses of microarray data and critically revised the manuscript. BdV provided support in immunohistochemical scoring and critically revised the manuscript. CHMJvE performed immunohistochemical staining, participated in data interpretation and critically revised the manuscript. MJB provided support for the data mining and critically revised the manuscript. KK provided surgical support in tissue acquisition and critically revised the manuscript. TA performed microarray analyses and critically revised the manuscript. HJMS participated in the conception and design of the study and critically revised the manuscript. VCTH participated in the conception and design of the study and critically revised the manuscript. PSH conceived and coordinated the study and critically revised the manuscript. All authors read and approved the final version of the manuscript.

## Supplementary Material

Additional file 1 **Table S1 showing the primer sequences used for qPCR validation**. Click here for file

Additional file 2 **Table S2 showing the 50 most significantly up-regulated genes after treatment**. Click here for file

Additional file 3 **Table S3 showing the 50 most significantly down-regulated genes after treatment**. Click here for file

Additional file 4 **Table S4 showing genes of the immune surveillance mechanism and their deregulation after treatment**. Click here for file
