# Supplementary material for: A randomised controlled phase II trial of pre-operative celecoxib treatment reveals anti-tumour transcriptional response in primary breast cancer
Source: Breast Cancer Res. 2013 Apr 8;15(2):R29. doi: 10.1186/bcr3409 (PMC3672758; doi:10.1186/bcr3409)
Supplement: Additional file 1 — Table S1 showing the primer sequences used for qPCR validation. [file bcr3409-S1.DOC]

**Supplementary table 1** Genes selected for qPCR analysis and their corresponding primers

| **Gene** | **Forward primer (5’ 🡪 3’)** | **Reverse primer (5’ 🡪 3’)** |
| --- | --- | --- |
| Target genes | |  |
| *ASPN* | tggaagagcctggacactaa | gagctttcggtttctgtctg |
| *CYR61* | aggacttattgggataca gcag | tgtgcttcatacaatgtcgtg |
| *GADD45A* | gccgaaagggttaatcatattt | accagtttattgtaggtattgtgtttc |
| *MARCKSL1* | catcatgggcagccagag | cacccttgggggataagtct |
| *MCM7* | agacaaggggcagacagcta | ttgacctgccagacattgag |
| *FOS* | ctccggtggtcacctgtact | gtcagaggaaggctcattgc |
| *COL1A1* | gagagcatgaccgatggatt | ccttcttgaggttgccagtc |
| *CDH11* | accccctgaaatcattcaca | tgggcagaaggtacaagtcc |
| Housekeeping genes | |  |
| *TBP* | cacgaaccacggcactgatt | ttttcttgctgccagtctggac |
| *RN18S1* | tcgaggccctgtaattggaa | ccctccaatggatcctcgtt |
