# Supplementary material for: A randomised controlled phase II trial of pre-operative celecoxib treatment reveals anti-tumour transcriptional response in primary breast cancer
Source: Breast Cancer Res. 2013 Apr 8;15(2):R29. doi: 10.1186/bcr3409 (PMC3672758; doi:10.1186/bcr3409)
Supplement: Additional file 2 — Table S2 showing the 50 most significantly up-regulated genes after treatment. [file bcr3409-S2.DOC]

**Supplementary table 2** List of significantly up-regulated genes after celecoxib treatment

| **Gene Symbol** | **Gene** | **Fold-change** | **95% CI** | **Description** |
| --- | --- | --- | --- | --- |
| FOS | ENSG00000170345 | 2.73 | 2.03-3.68 | Proto-oncogene protein c-fos |
| NA | ENSG00000235687 | 2.62 | 1.51-4.53 | Processed transcript (Havana) |
| CYR61 | ENSG00000142871 | 2.44 | 1.95-3.04 | Protein CYR61 precursor |
| ASPN | ENSG00000106819 | 2.36 | 1.64-3.39 | Asporin precursor |
| PIP | ENSG00000159763 | 2.20 | 1.24-3.90 | Prolactin-inducible protein precursor |
| RGS1 | ENSG00000090104 | 2.20 | 1.70-2.84 | Regulator of G-protein signaling 1 |
| C8orf4 | ENSG00000176907 | 2.19 | 1.50-3.20 | Uncharacterized protein C8orf4 |
| ANKRD30A | ENSG00000148513 | 2.12 | 1.29-3.47 | Ankyrin repeat domain-containing protein 30A |
| TFF1 | ENSG00000160182 | 2.00 | 1.13-3.56 | Trefoil factor 1 precursor |
| CTGF | ENSG00000118523 | 1.98 | 1.50-2.62 | Connective tissue growth factor precursor |
| GEM | ENSG00000164949 | 1.96 | 1.56-2.44 | GTP-binding protein GEM |
| EGR1 | ENSG00000120738 | 1.93 | 1.55-2.41 | Early growth response protein 1 |
| SFRP4 | ENSG00000106483 | 1.88 | 1.41-2.49 | Secreted frizzled-related protein 4 precursor |
| TNC | ENSG00000041982 | 1.87 | 1.35-2.60 | Tenascin precursor |
| GRP | ENSG00000134443 | 1.84 | 1.43-2.37 | Gastrin-releasing peptide precursor |
| SFRP2 | ENSG00000145423 | 1.83 | 1.37-2.44 | Secreted frizzled-related protein 2 precursor |
| COL12A1 | ENSG00000111799 | 1.79 | 1.40-2.29 | Collagen alpha-1(XII) chain precursor |
| COL5A1 | ENSG00000130635 | 1.77 | 1.40-2.24 | Collagen alpha-1(V) chain precursor |
| CDH11 | ENSG00000140937 | 1.76 | 1.41-2.20 | Cadherin-11 precursor |
| NID2 | ENSG00000087303 | 1.76 | 1.44-2.14 | Nidogen-2 precursor |
| RGS5 | ENSG00000143248 | 1.75 | 1.49-2.06 | Regulator of G-protein signaling 5 |
| PLK2 | ENSG00000145632 | 1.74 | 1.37-120 | Serine/threonine-protein kinase PLK2 |
| CRISPLD2 | ENSG00000103196 | 1.73 | 1.39-2.15 | Cysteine-rich secretory protein LCCL domain-containing 2 |
| COL5A2 | ENSG00000204262 | 1.72 | 1.34-2.22 | Collagen alpha-2(V) chain precursor |
| LRRC17 | ENSG00000128606 | 1.71 | 1.37-2.15 | Leucine-rich repeat-containing protein 17 precursor |
| COL1A1 | ENSG00000108821 | 1.71 | 1.33-2.19 | Collagen alpha-1(I) chain precursor |
| FAP | ENSG00000078098 | 1.70 | 1.38-2.10 | Seprase (Fibroblast activation protein) |
| SGK | ENSG00000118515 | 1.70 | 1.43-2.03 | Serine/threonine-protein kinase |
| IGF2 | ENSG00000167244 | 1.69 | 1.38-2.07 | Insulin-like growth factor II precursor |
| HMCN1 | ENSG00000143341 | 1.69 | 1.36-2.08 | Hemicentin-1 precursor (Fibulin-6) |
| WISP2 | ENSG00000064205 | 1.68 | 1.32-2.13 | WNT1-inducible-signaling pathway protein 2 precursor |
| EGR2 | ENSG00000122877 | 1.67 | 1.40-2.01 | Early growth response protein 2 |
| PDGFRL | ENSG00000104213 | 1.67 | 1.40-2.00 | Platelet-derived growth factor receptor-like protein |
| ITGBL1 | ENSG00000198542 | 1.67 | 1.36-2.05 | Integrin beta-like 1 |
| CPA3 | ENSG00000163751 | 1.66 | 1.26-2.19 | Mast cell carboxypeptidase A precursor |
| DCN | ENSG00000011465 | 1.66 | 1.27-2.17 | Decorin precursor |
| ZFP36 | ENSG00000128016 | 1.66 | 1.40-1.96 | Tristetraproline |
| FNDC1 | ENSG00000164694 | 1.65 | 1.28-2.13 | Fibronectin type III domain containing 1 |
| GLT8D2 | ENSG00000120820 | 1.65 | 1.34-2.02 | Glycosyltransferase 8 domain-containing protein 2 |
| MMP2 | ENSG00000087245 | 1.63 | 1.28-2.09 | 72 kDa type IV collagenase precursor |
| OGN | ENSG00000106809 | 1.63 | 1.23-2.16 | Mimecan precursor (Osteoglycin) |
| NTN4 | ENSG00000074527 | 1.62 | 1.12-2.34 | Netrin-4 precursor (Beta-netrin) |
| CTSK | ENSG00000143387 | 1.62 | 1.28-2.05 | Cathepsin K precursor |
| CSPG2 | ENSG00000038427 | 1.62 | 1.31-1.99 | Versican core protein precursor |
| SNAI2 | ENSG00000019549 | 1.62 | 1.35-1.93 | Zinc finger protein SLUG |
| AGR3_HUMAN | ENSG00000173467 | 1.62 | 1.03-2.54 | Anterior gradient protein 3 homolog precursor |
| SERPINE1 | ENSG00000106366 | 1.62 | 1.34-1.95 | Plasminogen activator inhibitor 1 precursor |
| AEBP1 | ENSG00000106624 | 1.61 | 1.26-2.05 | Adipocyte enhancer binding protein 1 precursor |
| IGJ | ENSG00000132465 | 1.60 | 1.02-2.53 | Immunoglobulin J chain |
| HTRA1 | ENSG00000166033 | 1.60 | 1.28-2.02 | Serine protease HTRA1 precursor |

NA, not available. CI, confidence interval.
