# Supplementary material for: A randomised controlled phase II trial of pre-operative celecoxib treatment reveals anti-tumour transcriptional response in primary breast cancer
Source: Breast Cancer Res. 2013 Apr 8;15(2):R29. doi: 10.1186/bcr3409 (PMC3672758; doi:10.1186/bcr3409)
Supplement: Additional file 3 — Table S3 showing the 50 most significantly down-regulated genes after treatment. [file bcr3409-S3.DOC]

**Supplementary table 3** List of significantly down-regulated genes after celecoxib treatment

| **Gene Symbol** | **Gene** | **Fold-change** | **95% CI** | **Description** |
| --- | --- | --- | --- | --- |
| HBD | ENSG00000223609 | 0.31 | 0.19-0.48 | Hemoglobin, delta |
| hsa-mir-29c | ENSG00000207966 | 0.62 | 0.49-0.78 | hsa-mir-29c |
| PROM1 | ENSG00000007062 | 0.64 | 0.43-0.95 | Prominin-1 precursor |
| CDC20 | ENSG00000117399 | 0.64 | 0.50-0.82 | Cell division cycle protein 20 homolog (p55CDC) |
| AKR1C2 | ENSG00000151632 | 0.67 | 0.49-0.93 | Aldo-keto reductase family 1 member C2 |
| SLC39A4 | ENSG00000147804 | 0.68 | 0.56-0.81 | Zinc transporter ZIP4 precursor |
| S100A9 | ENSG00000163220 | 0.68 | 0.47-0.97 | S100 calcium-binding protein A9 |
| SLPI | ENSG00000124107 | 0.69 | 0.48-0.98 | Antileukoproteinase 1 precursor (ALP) |
| UBE2C | ENSG00000175063 | 0.69 | 0.55-0.86 | Ubiquitin-conjugating enzyme E2 C |
| DGAT2 | ENSG00000062282 | 0.69 | 0.57-0.84 | Diacylglycerol O-acyltransferase 2 |
| LAPTM4B | ENSG00000104341 | 0.71 | 0.58-0.86 | Lysosomal-associated transmembrane protein 4B |
| MARCKSL1 | ENSG00000175130 | 0.71 | 0.62-0.82 | MARCKS-related protein |
| GINS1 | ENSG00000101003 | 0.72 | 0.60-0.86 | DNA replication complex GINS protein PSF1 |
| P11388-2 | ENSG00000131747 | 0.72 | 0.58-0.91 | TOP2A_HUMAN Isoform 2 of P11388 |
| PLIN | ENSG00000166819 | 0.73 | 0.56-0.95 | Perilipin (PERI) |
| ATAD2 | ENSG00000156802 | 0.73 | 0.61-0.88 | ATPase family AAA domain-containing protein 2 |
| HIST1H4C | ENSG00000197061 | 0.74 | 0.62-0.88 | Histone H4 |
| GPAM | ENSG00000119927 | 0.74 | 0.61-0.90 | Glycerol-3-phosphate acyltransferase mitochondria |
| THRSP | ENSG00000151365 | 0.74 | 0.57-0.97 | Thyroid hormone-inducible hepatic protein |
| MRPL48 | ENSG00000175581 | 0.74 | 0.64-0.86 | 39S ribosomal protein L48 mitochondrial precursor |
| POLR2K | ENSG00000147669 | 0.74 | 0.64-0.86 | DNA-directed RNA polymerases I II and III 7.0 kDa |
| IMPA2 | ENSG00000141401 | 0.74 | 0.60-0.92 | Inositol monophosphatase 2 |
| DHCR24 | ENSG00000116133 | 0.76 | 0.61-0.93 | 24-dehydrocholesterol reductase precursor |
| TFRC | ENSG00000072274 | 0.76 | 0.62-0.93 | Transferrin receptor protein 1 |
| PTDSS1 | ENSG00000156471 | 0.76 | 0.68-0.86 | Phosphatidylserine synthase 1 |
| G0S2 | ENSG00000123689 | 0.76 | 0.59-0.99 | Putative lymphocyte G0/G1 switch protein 2 |
| CRYAB | ENSG00000109846 | 0.76 | 0.59-0.99 | Alpha crystallin B chain |
| RACGAP1 | ENSG00000161800 | 0.77 | 0.64-0.92 | Rac GTPase-activating protein 1 |
| PTPRF | ENSG00000142949 | 0.77 | 0.67-0.89 | Receptor-type tyrosine-protein phosphatase F precursor |
| MEST | ENSG00000106484 | 0.77 | 0.66-0.90 | Mesoderm specific transcript isoform b |
| CLNS1A | ENSG00000074201 | 0.77 | 0.64-0.94 | Methylosome subunit pICln |
| CLDN3 | ENSG00000165215 | 0.78 | 0.63-0.96 | Claudin-3 |
| TACC2 | ENSG00000138162 | 0.78 | 0.64-0.94 | Transforming acidic coiled-coil-containing protein |
| TMEM99 | ENSG00000167920 | 0.78 | 0.67-0.90 | Protein TMEM99 precursor |
| BRF2 | ENSG00000104221 | 0.78 | 0.67-0.91 | RNA polymerase III transcription initiation factor, BRF1-like |
| CDC42EP4 | ENSG00000179604 | 0.78 | 0.73-0.84 | Cdc42 effector protein 4 |
| C11orf67 | ENSG00000087884 | 0.78 | 0.65-0.95 | UPF0366 protein C11orf67 |
| CYB561D1 | ENSG00000174151 | 0.78 | 0.73-0.84 | Cytochrome b561 domain-containing protein 1 |
| IER3IP1 | ENSG00000134049 | 0.78 | 0.68-0.90 | Immediate early response 3-interacting protein 1 |
| DHCR7 | ENSG00000172893 | 0.79 | 0.67-0.93 | 7-dehydrocholesterol reductase |
| RFC4 | ENSG00000163918 | 0.79 | 0.71-0.88 | Replication factor C subunit 4 |
| FOXC1 | ENSG00000054598 | 0.79 | 0.65-0.96 | Forkhead box protein C1 |
| YWHAZ | ENSG00000164924 | 0.79 | 0.68-0.91 | 14-3-3 protein zeta/delta |
| GRHL2 | ENSG00000083307 | 0.79 | 0.68-0.92 | Grainyhead-like protein 2 homolog |
| ENY2 | ENSG00000120533 | 0.79 | 0.67-0.93 | Enhancer of yellow 2 homolog |
| CENPF | ENSG00000117724 | 0.79 | 0.67-0.94 | Centromere protein F |
| PPFIA1 | ENSG00000131626 | 0.79 | 0.69-0.91 | Liprin-alpha-1 |
| MCM7 | ENSG00000166508 | 0.80 | 0.72-0.88 | DNA replication licensing factor MCM7 |
| RAD51AP1 | ENSG00000111247 | 0.80 | 0.69-0.92 | RAD51-associated protein 1 |
| EIF3S9 | ENSG00000106263 | 0.80 | 0.72-0.88 | Eukaryotic translation initiation factor 3 subunit 9 |

CI, confidence interval.
