# Supplementary material for: A randomised controlled phase II trial of pre-operative celecoxib treatment reveals anti-tumour transcriptional response in primary breast cancer
Source: Breast Cancer Res. 2013 Apr 8;15(2):R29. doi: 10.1186/bcr3409 (PMC3672758; doi:10.1186/bcr3409)
Supplement: Additional file 4 — Table S4 showing genes of the immune surveillance mechanism and their deregulation after treatment. [file bcr3409-S4.DOC]

**Supplementary table 4** Genes of immune surveillance and their deregulation after celecoxib treatment

| **Gene Symbol** | **Fold-change** | **95% CI** |
| --- | --- | --- |
| IgJ | 1.60 | 1.02 - 2.53 |
| HLA-DRα | 1.41 | 1.15 - 1.73 |
| HLA-DRβ2 | 1.36 | 1.11 - 1.67 |
| TLR2 | 1.32 | 1.18 - 1.48 |
| CD83 | 1.31 | 1.15 - 1.50 |
| HLA-DMα | 1.31 | 1.11 - 1.55 |
| CD14 | 1.28 | 1.08 - 1.52 |
| LY86 | 1.17 | 1.04 - 1.32 |
| CD74 | 1.16 | 1.05 - 1.29 |
| HLA-DQβ1 | 1.00 | - |
| CD48 | 1.00 | - |
| HLA-E | 1.00 | - |
| LGALS3 | 1.00 | - |
| TNFRSF8 (CD30) | 1.00 | - |
| MS4A1 (CD20) | 1.00 | - |
| CD19 | 1.00 | - |
| HLA-DOA | 1.00 | - |
| ARG1 | 1.00 | - |
| NOS2A | 1.00 | - |

CI, confidence interval.
